# An analysis of the accuracy of COVID-19 country transmission classification

**DOI:** 10.1038/s41598-022-13494-6

**Published:** 2022-06-10

**Authors:** I. Deza-Cruz, J. M. Prada, V. Del Rio Vilas

**Affiliations:** 1 Faculty of Health and Medical Sciences, University of Surrey, 30 Priestley Road, Surrey Research Park, Guildford, GU2 7YH Surrey UK; 2 World Health Organization, Regional Office for South-East Asia, World Health House, Indraprastha Estate, Mahatama Gandhi Marg, New Delhi, 110 002 India

**Keywords:** Public health, Epidemiology

## Abstract

Accurate epidemiological classification guidelines are essential to ensure implementation of adequate public health and social measures. Here, we investigate two frameworks, published in March 2020 and November 2020 by the World Health Organization (WHO) to categorise transmission risks of COVID-19 infection, and assess how well the countries’ self-reported classification tracked their underlying epidemiological situation. We used three modelling approaches: an ordinal longitudinal model, a proportional odds model and a machine learning One-Rule classification algorithm. We applied these models to 202 countries’ daily transmission classification and epidemiological data, and study classification accuracy over time for the period April 2020 to June 2021, when WHO stopped publishing country classifications. Overall, the first published WHO classification, purely qualitative, lacked accuracy. The incidence rate within the previous 14 days was the best predictor with an average accuracy throughout the period of study of 61.5%. However, when each week was assessed independently, the models returned predictive accuracies above 50% only in the first weeks of April 2020. In contrast, the second classification, quantitative in nature, increased significantly the accuracy of transmission labels, with values as high as 94%.

## Introduction

In the same month the novel coronavirus disease outbreak (COVID-19) became a global pandemic, the World Health Organization (WHO) released interim guidelines in March 2020 to help Member States (MS) assess the situation at national and sub-national levels^1^. This document established four transmission classes for COVID-19 based on the intensity of transmission: ‘no cases’, ‘sporadic cases’, ‘cluster of cases’, and ‘community transmission’ (Table 1). The guidance also provided key recommendations for the implementation of public health and social measures (PHSM). Since the release of the guidelines, the WHO has published the MS transmission classification, as informed by the countries’ relevant health authorities. A fifth ‘pending’ class was available when the MS did not report their classification to WHO. This classification system was used mostly at national level and was reviewed on a weekly basis by MS as new evidence became available. WHO publishes MS classifications in weekly situational reports together with the latest epidemiological evidence, namely, total confirmed cases and deaths to date, and total new cases and new deaths.

**Table 1:** Transmission classes of COVID-19 and criteria for each class published by WHO in March 2020 and in November 2020.

WHO interim guidance March 2020		WHO interim guidance November 2020	
Transmission classes	Criteria	Transmission classes	Criteria
No cases	No reported cases	No cases	No new cases detected for at least 28 days
Sporadic cases	One or more cases, imported or locally acquired	Sporadic cases	Cases detected in the past 14 days are all imported, sporadic or are all linked to imported/sporadic cases
Clusters of cases	Most cases of local transmission linked to chains of transmission	Clusters of cases	Cases detected in the past 14 days are predominantly limited to well-defined clusters that are not directly linked to imported cases
Community transmission	Outbreaks with the inability to relate confirmed cases through chains of transmission for a large number of cases, or by increasing positive tests through sentinel samples (routine systematic testing of respiratory samples from established laboratories Units: 1. Hospitalization: new COVID-19 hospitalizations per 100,000 population 2. Mortality: COVID-19 attributed deaths per 100,000 population per week averaged over a two-week period 3. Case incidence: new confirmed cases per 100,000 population per week averaged over a two-week period 4. Testing: test positivity proportion from sentinel sites averaged over a two-week period	CT1	Hospitalization rate: < 5 Mortality: < 1 Case incidence: < 20 Testing: < 2%
		CT2	Hospitalization rate: 5–10 Mortality: 1–2 Case incidence: 20–50 Testing: 2–5%
		CT3	Hospitalization rate: 10–30 Mortality: 2–5 Case incidence: 50–150 Testing: 5–20%
		CT4	Hospitalization rate: > 30 Mortality: > 5 Case incidence: > 150 Testing: > 20%

The classification criteria were updated in November 2020. The community transmission class was split into four new sub-classes (from level 1—CT1 to level 4—CT4) according to a set of criteria averaged over the latest two-week period: case incidence (new confirmed cases per 100,000 population per week), mortality (number of COVID-19 attributed deaths per 100,000 population per week), hospitalization rate (new COVID-19 hospitalizations per 100,000 population per week), and proportion of positive tests (test positivity proportion from sentinel sites), Table 1^2^. The original classification from March was qualitative, but the new classification provided quantitative thresholds for each criterion to characterise the four subclasses CT1-CT4, Table 1. Despite this substantial improvement, the new guidelines do not provide advice on how to aggregate the criteria values given by MS to reach one of the four new community transmission sub-classes (we note here that the key changes in the November 2020 guidelines mainly affected the community transmission class). In the absence of such advice, multiple combinations at the discretion of MS are possible. For example, a country could claim to have less than 5 hospitalizations per 100,000 (and hence being classified as CT-1), a mortality of > 5 deaths per 100,000 (and hence being classified as CT-4), between 20 and 50 new cases per 100,000 (and hence being classified as CT-2), and a test positivity of 5% to 20% (leading to a classification as CT-3) (Table 1 in Supplementary Material). Such discretion would limit comparisons of transmission status and of interventions effectiveness among MS, and might put into question travel restrictions on third countries based on their transmission classification.

The relevance of accurate transmission classification cannot be overstated. The decision to implement any control measures, and necessary spatial and temporal adaptations to best suit the ongoing epidemiology of the outbreak, must be informed with an unbiased understanding of the transmission in the community. This is particularly clear for PHSM which have shown to limit transmission of COVID-19. WHO has recognised this and published recent guidelines to calibrate PHSM deployment^1,2^.

Here, we investigate the criteria established by WHO to categorise the risk of COVID-19 infection at country level and explore the robustness of the transmission classes and sub-classes (for the latest guidelines pertaining to community transmission) as reported by MS. Specifically, we assess (1) the predictability of the two guidelines (published in March 2020, and November 2020) over time against the standard epidemiological data published weekly by WHO, and (2) the sources of ambiguity in the application of the November 2020 classification.

## Materials and methods

### Materials

Country-specific epidemiological data was imported from the WHO website through the package COVID-19^3^ (https://data.humdata.org/dataset/coronavirus-covid-19-cases-and-deaths) for the period 6 April 2020 to 13 June 2021, inclusive. The following variables were selected for analysis: number of confirmed cases, deaths, and hospitalizations; number of patients in ventilator; number of patients in intensive care units (ICU), and country population. Data for the same period on the self-reported country transmission classification^1^ was downloaded from WHO’s published situational reports and aggregated by week. To date no country classification following the new guidelines proposed by WHO in November 2020^2^ has been published.

### Methods

#### Assessment of the predictability of classification guidelines

We explored three distinct modelling approaches to assess the predictability of the reported country classifications, for both the initial classification from March 2020^1^ and the updated classification from November 2020^2^. Firstly, an ordinal longitudinal regression model^4^ was fitted to estimate the probability that a country *i* at the observation time *j* were classified into the ordinal category *k* (country classification) using the R package ‘mixor’^5^. Longitudinal models allow the modelling of time dependence between observations in the time series. The variable ‘country’ was identified as the clustering factor and the different predictors were introduced in the model one at a time. A second model was implemented considering each week independently: a proportional odds logistic regression model in a Bayesian framework through the R package “runjags”^6^. The proportional odds model is a class of generalized linear models used for modelling the dependence of an ordinal response on discrete or continuous covariates^7^. The Bayesian approach can estimate the posterior distribution of the response parameter, quantifying the probability of its expected classification label^8^. For both the ordinal regression model and the proportional odds logistic model, we considered univariate models first, then extended those to bivariate models adjusting for the variable with lowest deviance in the univariate models. Further inclusion of additional variables beyond bivariate models was not considered due to collinearity among predictors. Our final approach was a machine learning model adopting the One-Rule algorithm that classified examples on the basis of a single attribute and then selects the rule with the smallest total error^9^. Despite its simplicity, this machine learning algorithm has high accuracy and is a parsimonious alternative to systems that learn more complex rules^9^. The model was implemented using the R package “OneR”, which uses the One-Rule classification algorithm with enhancements for sophisticated handling of numeric data and missing values^10^. Further details of the models are included in the Supplementary Material.

When analysing the initial classification published by WHO in March 2020, only observations from countries that were labelled within one of the three epidemiological categories (i.e., ‘sporadic cases’, ‘clusters of cases’ or ‘community transmission’) were used; countries under the class ‘pending’ were ignored (see below). Also, due to the lack of instructions in the guidelines as to which epidemiological indicator was chosen to infer the transmission label, the following variables (cases, deaths, hospitalizations, ventilators and ICU) were explored. The average daily values for the previous 7, 14 and 21 days were calculated for each variable in addition to the ratio per 100,000 population.

For the country classification guidelines published in November 2020^2^, we assessed the possible approaches that countries might follow to combine values across the criteria and how scores are aggregated to reach the overall label. We explored the median of the individual criteria score (i.e., the most common label, CT: 1–4), the mean score (CT: 1–4), and the maximum score for any criterion (i.e., the greatest of label CT: 1–4 for any criteria), and attempted to recover them through the three different models. Following the instructions in the November 2020 guidelines, we used the average of daily new cases in the previous 14 days, mortality rate and hospitalization rate to inform the countries’ categorization and excluded testing rate as it was not readily available for the majority of observations.

Each of the variables on the complete dataset were standardised by subtracting the mean and dividing by the standard deviation so that each variable ended with mean 0 and standard deviation 1. The complete data set was divided into a 75–25% training set and test set, respectively. Due to the high variability of the results with the One-Rule Machine Learning model depending on the data set split, we report the average of 400 bootstrap replicates. The accuracy of the models was calculated by creating a confusion matrix (Supplementary material) that compared the number of correct predictions in the test dataset for each model. Additionally, to quantify the statistical significance of the difference between the accuracies of the initial and new classifications, McNemar tests were performed.

#### Prediction of pending country classifications

When MS delayed submitting their classification to WHO, a label ‘pending’ was assigned. The epidemiological transmission situation was unknown. The above models were used to predict the transmission classification of the countries pending classification in one of the three transmission categories, as per March 2020 guidelines.

## Results

### Descriptive statistics of classification groups

**Figure 1:**
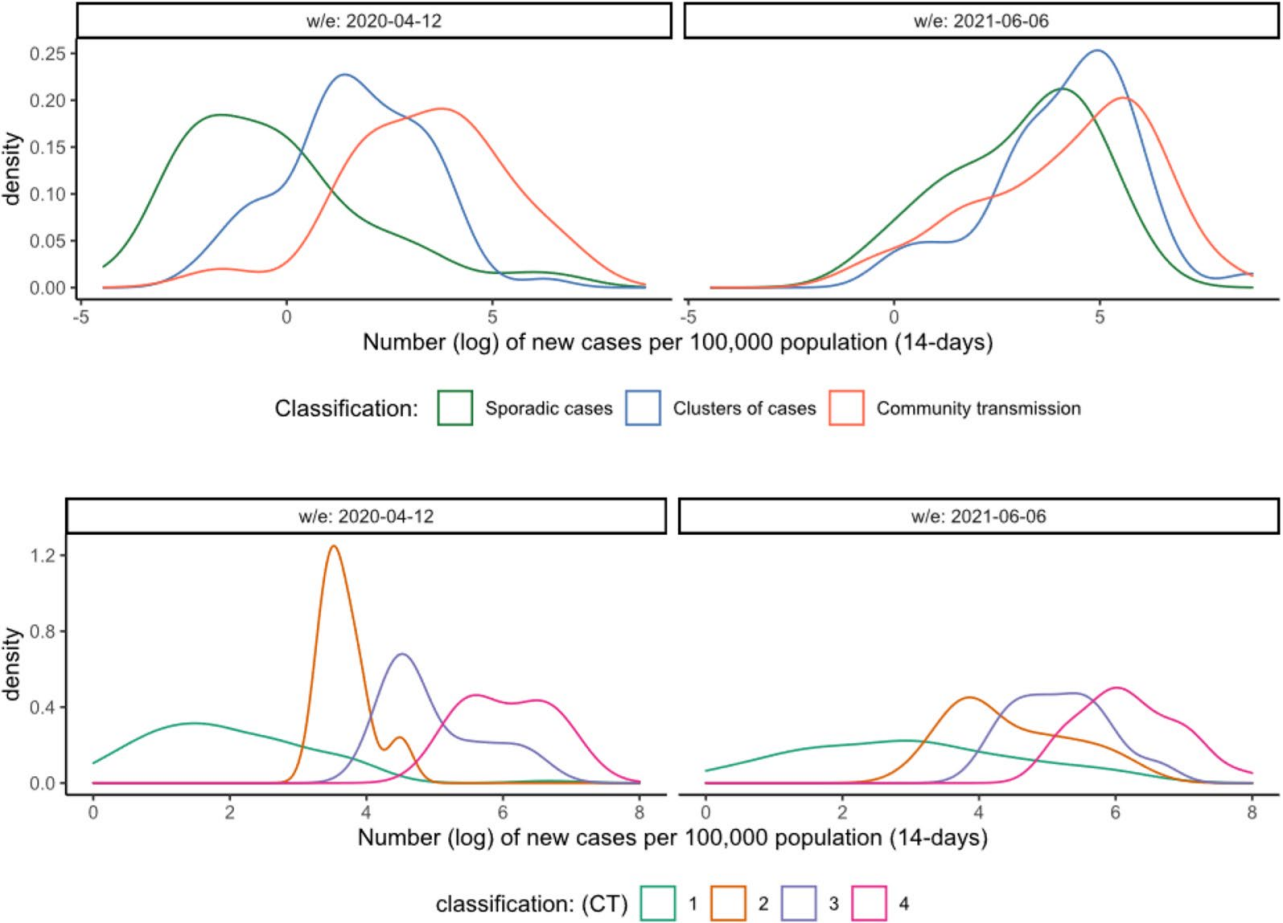
Density of weekly incidence rate per 100,000 population (14 days average) in each transmission class. Top row: following classification guidance published in March 2020. Bottom row: following classification guidance published in November 2020 and the median of scores as the aggregate approach. The overlap between the different categories (top right) illustrates the inconsistencies in the initial classification (i.e., countries reported similar number of new cases but different categories). The bottom row shows distinctive grouping of the data around each category indicating a consistent classification through time. The complete distribution series can be found in the supplementary material.

The complete data set included information on 202 countries collected for 61 weeks between 6 April 2020 and 13 June 2021, inclusive. Following guidance published in March 2020, each country submitted to WHO their own classification category, as either ‘Sporadic cases’, ‘Clusters of cases’ or ‘Community transmission’ according to their national level of transmission. First, we assessed patterns that could indicate distinctive grouping of the data around the three initial categories and found that the number of new cases achieved better separation between categories than other epidemiological indicators such as number of deaths or hospitalizations. The clearest divisions between categories could be seen with the log number of new cases per 100,000 population (14-day average) in the first weeks of implementing this classification in April 2020 (Fig. 1, top left). Discrimination is however inconsistent throughout the study period and in most weeks the different categories overlap (Fig. 1, top right). This occurs when countries reported similar incidence rates, but classified themselves in different categories. In contrast, a retrospective application of the transmission classification guidelines published in November 2020, shows clear divisions between categories for the log number of new cases per 100,000 population (14-day average) using the median of scores as the aggregate approach (Fig. 1, bottom). This consistency was observed regardless of the aggregate criteria to reach the overall situational level (e.g., ‘median’, ‘mean’ or ‘greatest’ score). An effective classification system with clearly defined categories would show patterns with distinctive grouping of the data around each category.

**Figure 2:**
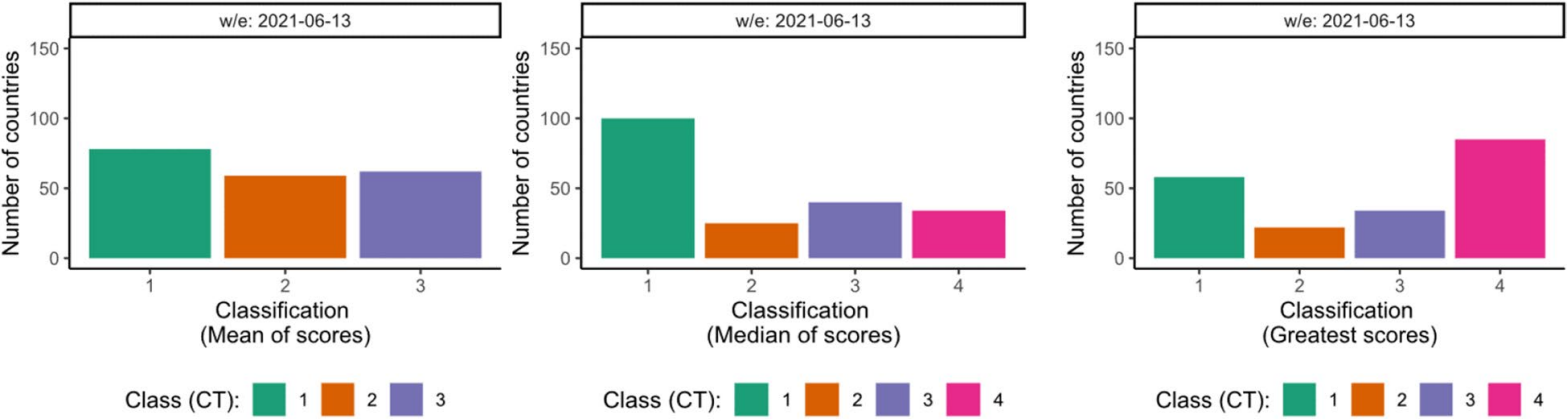
Frequency of country distribution according to the aggregate approach for the new classification guidelines published in November 2020. Left: Mean of scores. Centre: Median of scores. Right: Greatest of the scores. The complete distribution can be found in the supplementary material.

Although the updated classification guidelines published in November 2020 established a defined criteria to categorise transmission into four sub-classes (CT1-4), the resulting sub-class varied depending on which overall aggregate approach was used, e.g., the mean of the epidemiological criteria scores, the median or the greatest score. This caveat is illustrated in Fig. 2. Applying this guidance on the data reported by MSs, on the week ending on 13 June 2021, 85 (42.1%) countries would be classified as CT4, the highest transmission class, if the greatest criterion score was used as the overall aggregate rule. This compares with no countries classified as CT4 when the mean of all the criteria scores is used. The situation in the former scenario occurs when countries reported a case incidence rate greater than 150 cases (CT4), a mortality rate smaller than 1 death (CT1) and no hospitalizations (CT1) averaged within the previous two weeks. Whereas in the latter scenario, a number of countries reported an incidence rate greater than 150 cases (CT4), a mortality rate greater than 15 deaths (CT4) but a hospitalization rate smaller than 5 (CT1). Similarly, the number of countries in the lowest transmission sub-class CT1 would also change depending on the aggregate approach: 78 (38.6%), 100 (49.5%) and 58 (28.7%) countries would be in CT1 class if using the mean of the scores, the median or the greatest score, respectively, Fig. 2.

### Accuracy of classification guidelines

Overall, the models implementing the initial classification released in March 2020 showed poor accuracy (Table 2). The best performing model, the One-Rule Machine Learning algorithm, had an overall accuracy of 62.9% (59.0–67.5%) after 400 bootstrap repetitions, thus suggesting that for 37.1% of the countries any single data stream did not support their self-reported classification. The other two models, namely, the mixed effects ordinal longitudinal regression and the proportional odds logistic regression, only reached accuracies of 59.2% and 32.8%, respectively. Also, there were discrepancies regarding the best predictors for each model. The One-Rule Machine Learning only allows one predictor, and the number of new cases in the previous 14 days resulted in the model with the greatest accuracy. In contrast, the best ordinal longitudinal regression models included two predictors: new hospitalizations per 100,000 population in the previous 14 days and the number of new cases per 100,000 in the previous 14 days. Alternatively, the proportional odds logistic regression model included the number of new cases per 100,000 population in the previous 14 days and the number of new patients on ventilator in the previous 21 days, Table 2.

**Table 2:** Summary of accuracy for best fitting predictors across the three models considered.

Methodology	Initial classification (March 2020)		New classification (November 2020)	
	Predictors	Accuracy (95% CI)^a^	Predictors	Accuracy (95% CI)^a^
Ordinal longitudinal regression	New deaths per 100 k (7-days)	0.591 (0.573–0.601)	New deaths per 100 k (14 days) [Median score]^b^	0.782 (0.767–0.797)
	New hosp. (14-days) New cases per 100 k (14-days)	0.592 (0.574–0.610)	New deaths. per 100 k (14 days) New cases per 100 k (14 days) [Median score]^b^	0.823 (0.809–0.837)
Proportional odds model (Bayesian)	New cases (14-days)	0.257 (0.144–0.405)	New deaths per 100 k (14 days) [Median score]^b^	0.737 (0.721–0.754)
	New cases per 100 k (14-days) New patients on ventilator (21-days)	0.328 (0.312–0.346)	New cases per 100 k (14 days) New deaths per 100 k (14 days) [Median score]^b^	0.750 (0.733–0.796)
One-rule classification algorithm	New cases (14-days)	0.629 (0.590–0.675)	New cases per 100 k (14 days) [Greatest score]^b^	0.943 (0.933–0.951)

^a^Refers to total accuracy for the ordinal longitudinal regression and average accuracy for the other two models (April 2020 to June 2021).

^b^Aggregate criteria approach to summarise the sub-classes CT-1 to CT-4.

**Figure 3:**
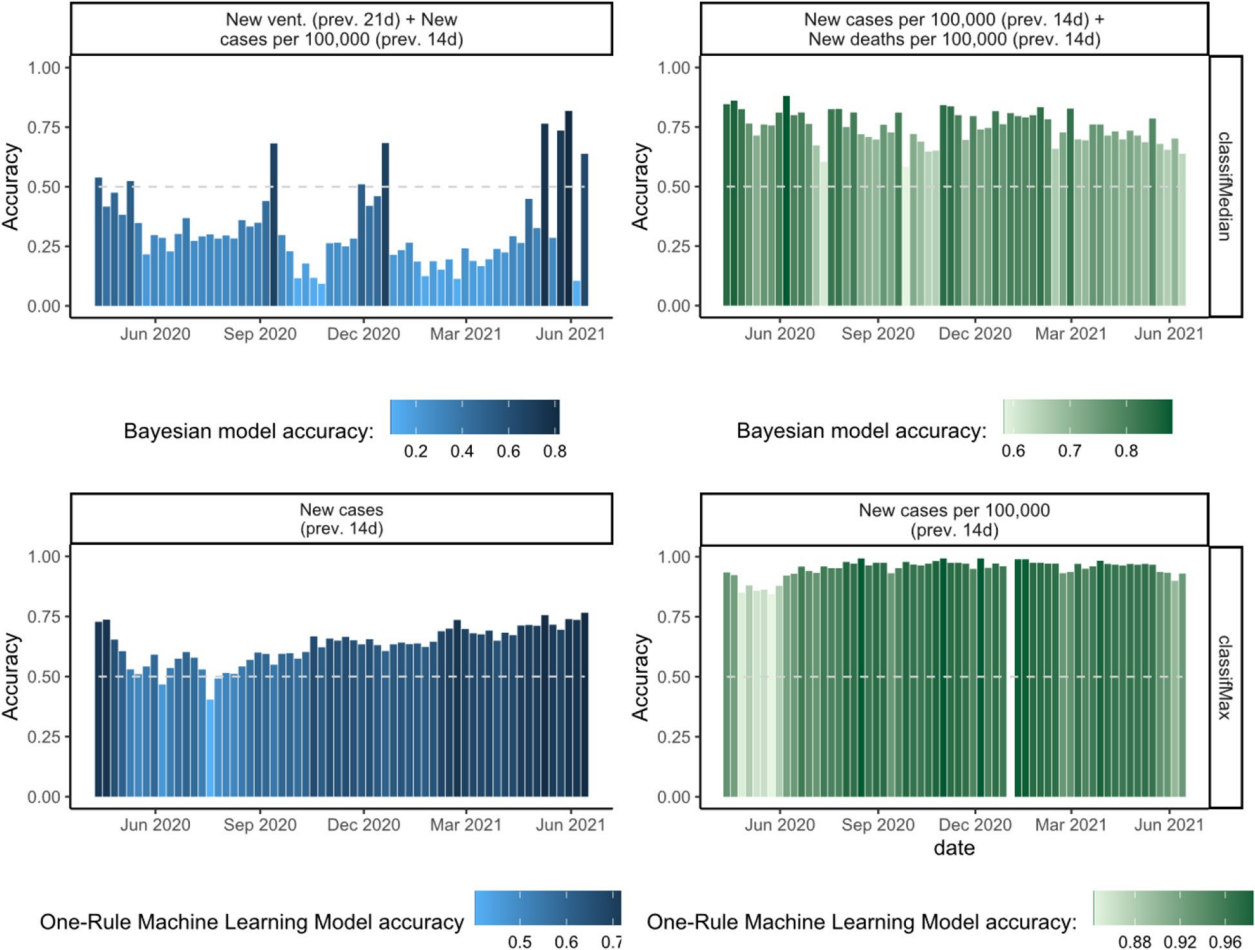
Predictive accuracies of best performing models by week. Each bar represents one week. The predictors for each model are detailed on the top of each graph. Left column: initial classification from March 2020. Right column: updated classification from November 2020. Top row: Proportional Bayesian Odds models. Bottom row: One-Rule Machine Learning models.

In addition, the One-Rule machine learning and Bayesian model allowed exploring predictions of transmissions labels for each week independently. Both models showed a clear trend similar to that observed in the visual assessment. The accuracy of the classification was adequate in the initial weeks of April 2020 (peaks of 78% and 72% for the One-Rule algorithm and Bayesian model, respectively). However, the accuracy of the classifications decreased substantially towards the end of the study period with numerous weeks below 50% accuracy suggesting that the criteria for classification was applied inconsistently throughout time (Fig. 3, top left panel). The complete set of model accuracies and best predictors can be found in the supplementary material.

The accuracy of the models implementing the updated classification released in November 2020 was good overall (Table 2). The best performing model was again the One-Rule Machine Learning algorithm reaching an overall accuracy of 94.3% (93.3–95.1%, *p* value < 0.001). The predictor for this model was the number of new cases per 100,000 population (14-days average) and the aggregate approach was the ‘greatest of scores’. The weekly accuracies were above 85% (Fig. 3). Other predictors such as the mortality rate (14-days average) when categorising countries into a CT1-4 category by the median score showed similar accuracy (93.6%). Categorising countries by the mean score had a slightly lower predictive accuracy of 86.2% when considering the incidence rate and 85.2% when considering the mortality rate.

The ordinal longitudinal regression model and the proportional odds logistic regression model reached accuracies as high as 82.3% and 75.0%, respectively. Although the best model followed the ‘greatest score’ approach, all of the remaining best performing models followed the ‘median score’ as the aggregate labelling approach (Table 2). The predictors associated with the best models included the number of new cases per 100,000 population and new deaths per 100,000 population both in the previous 14 days. Additionally, these models had a consistently high accuracy throughout the observed period (Fig. 3). As with previous algorithms, the number of hospitalizations was a poor predictor and this predictor only appeared in one of the overall best ten models (Table 2).

### Prediction of pending classifications

There were 303 occurrences where countries were classified as ‘pending’ and no information about their transmission status was available. The models with the greatest accuracy were used to predict the categories of those countries; however, the disagreement between the model predictions was high, suggesting the difficulty of implementing the initial classification criteria onto new data.

No country was predicted in the same category by all three models and 30% (91 out of 303 observations) were classified in a different category by each model (Supplementary material). The greatest agreement in predicted outcomes was achieved between the Bayesian and One-Rule machine learning models with 62.0% agreed classifications (188 out 303 instances). The mixed effect ordinal longitudinal models were in agreement with any other models on 18 occasions (5.9%) only. This is not entirely unexpected as these models perform similarly with large samples but can diverge when analysing smaller data sets such as each week independently.

## Discussion

We have analysed the reliability of two COVID-19 country classification guidelines by implementing three distinct modelling approaches. Overall, our results indicate that implementing the updated classification of November 2020 would have achieved greater predictive accuracy (94.3%, *p* value < 0.001), which is consistent over time, and reflects the epidemiological situation of COVID-19 transmission in a particular country more accurately than the initial transmission classification guidance for which models only reached 62.9% accuracy.

In the best-case scenario, using the March 2020 classification, our models showed that for three out of four countries the data supported their self-reported transmission labels. However, this situation was only valid for three weeks out of the total count of 61 weeks under study (4.9%). In the worst-case scenario, the models with the lowest accuracy showed that only in one out of three countries the data supported their self-reported transmission labels. If implementation of PSHM was informed by the initial classification, most of the countries, most of the time, would have deployed insufficient measures.

The guidance released with the updated classification in November 2020 has some major advantages compared with the classification published in March 2020. The updated guidelines propose some primary epidemiological indicators and specific criteria to assess the level of COVID-19 community transmission. These objective criteria reduce inaccuracies in the categorisation of the overall situational label, but it is unclear how to aggregate the different data streams considered. Aggregating the epidemiological indicators by the greatest of the scores will return a different overall CT transmission label than aggregating the indicators by the mean of the scores or the median. Our models that used the greatest score to inform the overall label performed slightly better (94.6% accuracy) than the models that used the median score (93.2% accuracy). When using the greatest score, the best predictor is new cases in 14 days per 100,000. This suggests that this data stream is the most “extreme” (i.e. contains many high values) compared to the other ones—and thus the thresholds defined by WHO might be slightly lower than those of other predictors, such as deaths. If using the median score, the best predictor variable is the new deaths in 14 days per 100,000.

There were several limitations in our study. Our models were created before the availability of vaccines and the vaccination programmes were rolled out in most countries. Extensive studies have demonstrated the effectiveness of vaccinations in decreasing the risk of severe COVID-19 complications and fatalities^11–14^. The introduction of vaccinations and other non-pharmaceutical interventions may affect the accuracy of models based on predictors related to the number of hospitalizations or deaths^15^. Additionally, the variability in the data reported by national health authorities has been highlighted as a concern^16^. For instance, some countries reported low number of hospitalizations but high numbers of deaths. Although it is not the aim of our manuscript to analyse the disparity between these figures, the low number of hospitalizations with regards to such high number of deaths may be explained by lack of access to healthcare in a particular country or poor quality of data collection and reporting. In other cases, deaths could happen in the community, at home or at nursing homes. Also, many of these instances happened in the first weeks of the pandemic when testing was not widely available in many countries. Consequently, the test positivity rate per country, one of the predictors included to inform the transmission classification in the updated criteria in November 2020, was not readily available and therefore not included in the analysis.

Using either crude values or population ratios can distort results. Small countries will show higher ratios per 100,000 population with lower crude values than large countries. In our analysis we considered both types of metrics and the better performing models included only predictors per 100,000 population.

To our knowledge, this is the first study of the validity of transmission classification labels. There is an ongoing stream of ever more sophisticated frameworks to classify or rank countries in their efforts to control the epidemic. This clashes with the reality in many countries where the data they generate cannot populate with any reliability such frameworks. Our results highlight the need for accurate and precise guidelines that reflect the actual status of transmission of diseases in a worldwide context. This will allow for adequate comparisons between countries and help national and international health authorities to establish adequate control measures.

## Supplementary Information

Supplementary Information.
