# Supplementary material for: An analysis of the accuracy of COVID-19 country transmission classification
Source: Sci Rep. 2022 Jun 10;12:9604. doi: 10.1038/s41598-022-13494-6 (PMC9186008; doi:10.1038/s41598-022-13494-6)
Supplement: Supplementary file 1 — Supplementary Information. [file 41598_2022_13494_MOESM1_ESM.docx]

**Supplementary material:**

Table 1. Summary of countries and their different classifications (CT) according to the number of cases, hospitalizations or deaths following the new guidance published in November 2020. This example illustrates the uncertainty derived by the lack of instructions on the application of the guidance.

| Country | Week ending | New cases per 100k (14-days) | New hosp. per 100k (14-days) | New deaths per 100k (14-days) | Classif. according to cases (CT) | Classif. according to hosp.  (CT) | Classif. according to deaths (CT) |
| --- | --- | --- | --- | --- | --- | --- | --- |
| Antigua and Barbuda | 19/04/2020 | 22.8 | 0.0 | 5.2 | 2 | 1 | 4 |
| Andorra | 17/05/2020 | 39.0 | 0.0 | 18.2 | 2 | 1 | 4 |
| Bermuda | 17/05/2020 | 26.6 | 0.0 | 6.3 | 2 | 1 | 4 |
| Andorra | 24/05/2020 | 26.0 | 0.0 | 11.7 | 2 | 1 | 4 |
| Bermuda | 24/05/2020 | 36.0 | 0.0 | 6.3 | 2 | 1 | 4 |
| Mexico | 24/05/2020 | 47.0 | 0.0 | 5.5 | 2 | 1 | 4 |
| Netherlands | 24/05/2020 | 35.2 | 4.6 | 5.9 | 2 | 1 | 4 |
| Spain | 28/06/2020 | 20.4 | 0.0 | 5.1 | 2 | 1 | 4 |
| Bolivia | 25/10/2020 | 45.6 | 0.0 | 6.3 | 2 | 1 | 4 |
| Bolivia | 01/11/2020 | 38.2 | 0.0 | 5.2 | 2 | 1 | 4 |
| Wallis and Futuna | 16/05/2021 | 37.8 | 0.0 | 12.6 | 2 | 1 | 4 |
| Antigua and Barbuda | 23/05/2021 | 36.4 | 0.0 | 11.4 | 2 | 1 | 4 |
| Antigua and Barbuda | 30/05/2021 | 45.7 | 0.0 | 19.7 | 2 | 1 | 4 |
| Antigua and Barbuda | 06/06/2021 | 24.9 | 0.0 | 9.3 | 2 | 1 | 4 |
| North Macedonia | 06/06/2021 | 44.4 | 0.0 | 16.4 | 2 | 1 | 4 |
| North Macedonia | 13/06/2021 | 29.8 | 0.0 | 9.9 | 2 | 1 | 4 |

**Methods: description of models**

Firstly, an ordinal longitudinal regression model^5^ was fitted to estimate the probability *p_ijk_* that a country *i* at the observation time *j* were classified into the ordinal category *k* (country classification) using the R package ‘mixor’.^6^ As described elsewhere,^7^ the cumulative probability at *j* is P(*Y_ij_* ≤ *k*) = and the mixed-effects logistic regression model for the *K -1* cumulative logit is then given by:

|  | (1) |
| --- | --- |

where *c_k_* are the increasing thresholds, *x_ij_* is the variable vector, is the vector of regression parameters and *z_ij_* is the vector of random effects. The response vector *y_i_* = (*Y_ij1_*, …, *Y_ijK_*) is the classification outcome for country *i*.^8^ This approach was chosen as covariates may be at any level and do not have to follow the proportional odds assumption. It also allows fitting an ordinal response model when observations are collected longitudinally and account for the correlation between repeated measurements of a country by applying an adaptive Gauss-Hermite quadrature to numerically integrate over the distribution of random effects and Fisher scoring to obtain the likelihood solution.^8^ The main assumptions included that the response functioned followed a logit link which was tested by a likelihood ratio test and that the random effects were correlated. The variable ‘country’ was identified as the clustering factor and the different predictors were introduced in the model one at a time. We considered univariate models first, then extended those to bivariate models adjusting for the variable with lowest deviance in the univariate models. Further inclusion of additional variables beyond bivariate models was not considered, due to collinearity among predictors.

A second model was implemented considering each week independently: a proportional odds logistic regression model in a Bayesian framework through the R package “runjags”.^9^ The proportional odds model is a class of generalized linear models used for modelling the dependence of an ordinal response on discrete or continuous covariates^10^. The Bayesian approach can estimate the posterior distribution of the response parameter, quantifying not only its expected classification but also the associated uncertainty.^11^ We applied this model for each predictor independently. Following previous methodology,^12^ we calculated the probability *p_ik_* that a country *i* would be classified within a particular category *k* by inferring the latent thresholds *c_k_* and an unknown intercept *β*. The model can be described as:

| *y*_i_ ~ categorical(*p_i_*)  p_i1_ =1-γ_i_  *p_ik_ =* *γ*_ik-1_ - *γ*_ik_  logit(*γ*_ik_) = *β^.^x_i_ - c_k_* | (2) |
| --- | --- |

Two chains were run in parallel to draw 4000 posterior samples from each chain with no thinning after a burn-in of 1000 samples. Uninformative priors were chosen for the latent coefficients and convergence was assessed by visual assessment of the chains and the Gelman-Rubin statistic.^13^

Our final approach was a machine learning model adopting the One-Rule algorithm that classify examples on the basis of a single attribute and then selects the rule with the smallest total error.^14^ Despite is simplicity, this machine learning algorithm has high accuracy and is a parsimonious alternative to systems that learn more complex rules.^14^ The classification rules induced by machine learning systems are judged by two criteria: their classification accuracy on a test set and their complexity, and require no assumptions about the distribution of related data.^14^.As described previously,^14^ the algorithm starts counting frequencies in the training data set of value *v* within a particular category *k* for a given attribute *A* (e.g. number of cases). Next, for each numerical attribute, a nominal version is created by defining finite intervals of values where these intervals become the values of a nominal version of *A*. For each nominal version of the attribute *A_j_* and its value *v_j_*, an optimal class with the greatest frequency of data instances is created that satisfy the condition *A_j_* = *v_j_*. After the inference of the rules, error rates are computed, and the attribute which has the lowest total error rate is chosen as the best attribute.^15^ The model was implemented using the R package “OneR”, which uses the One-Rule classification algorithm with enhancements for sophisticated handling of numeric data and missing values.^16^


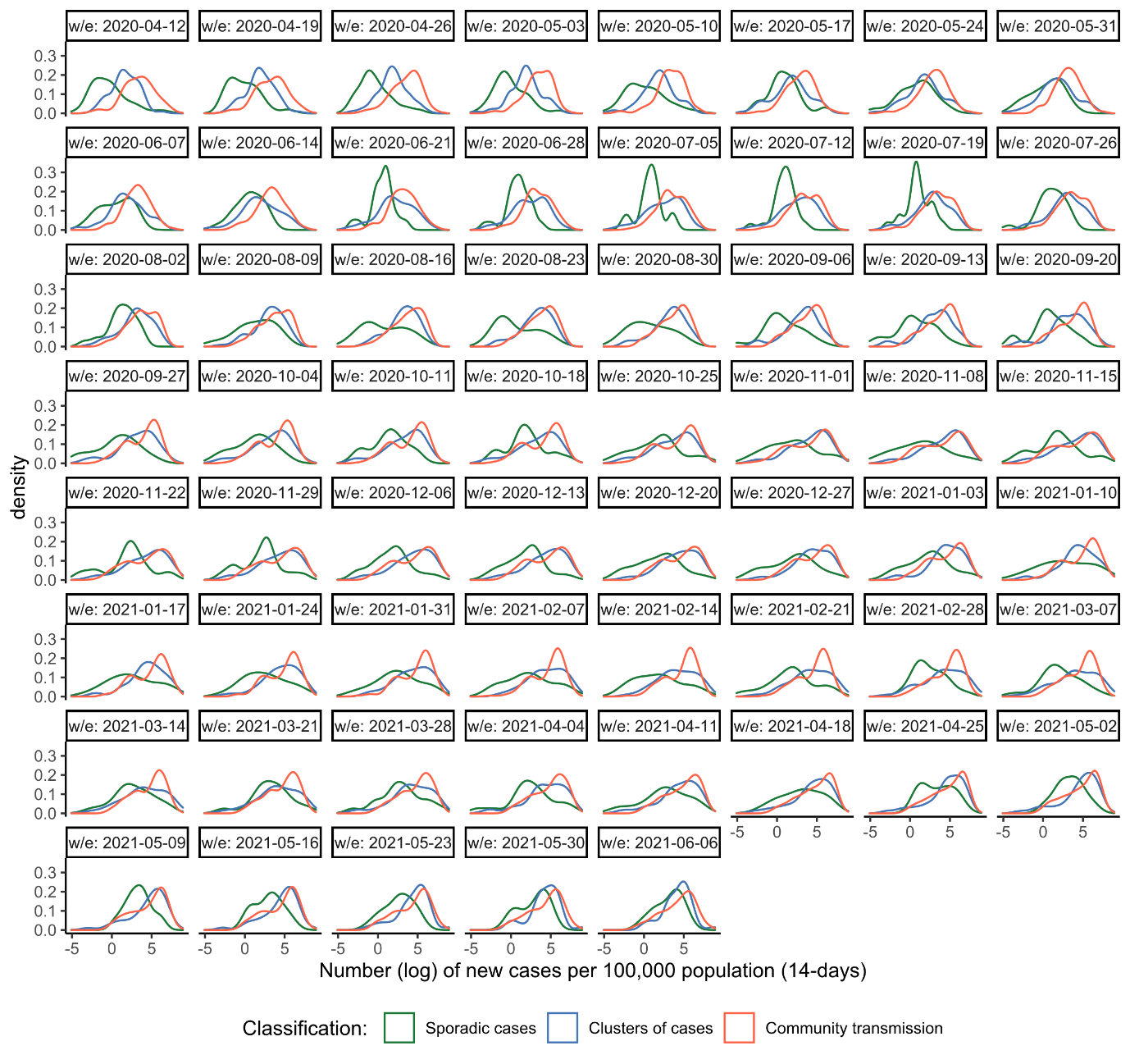


Figure 1. Density of weekly incidence rate per 100,000 population (14 days average) in each transmission class following classification guidance published in March 2020.


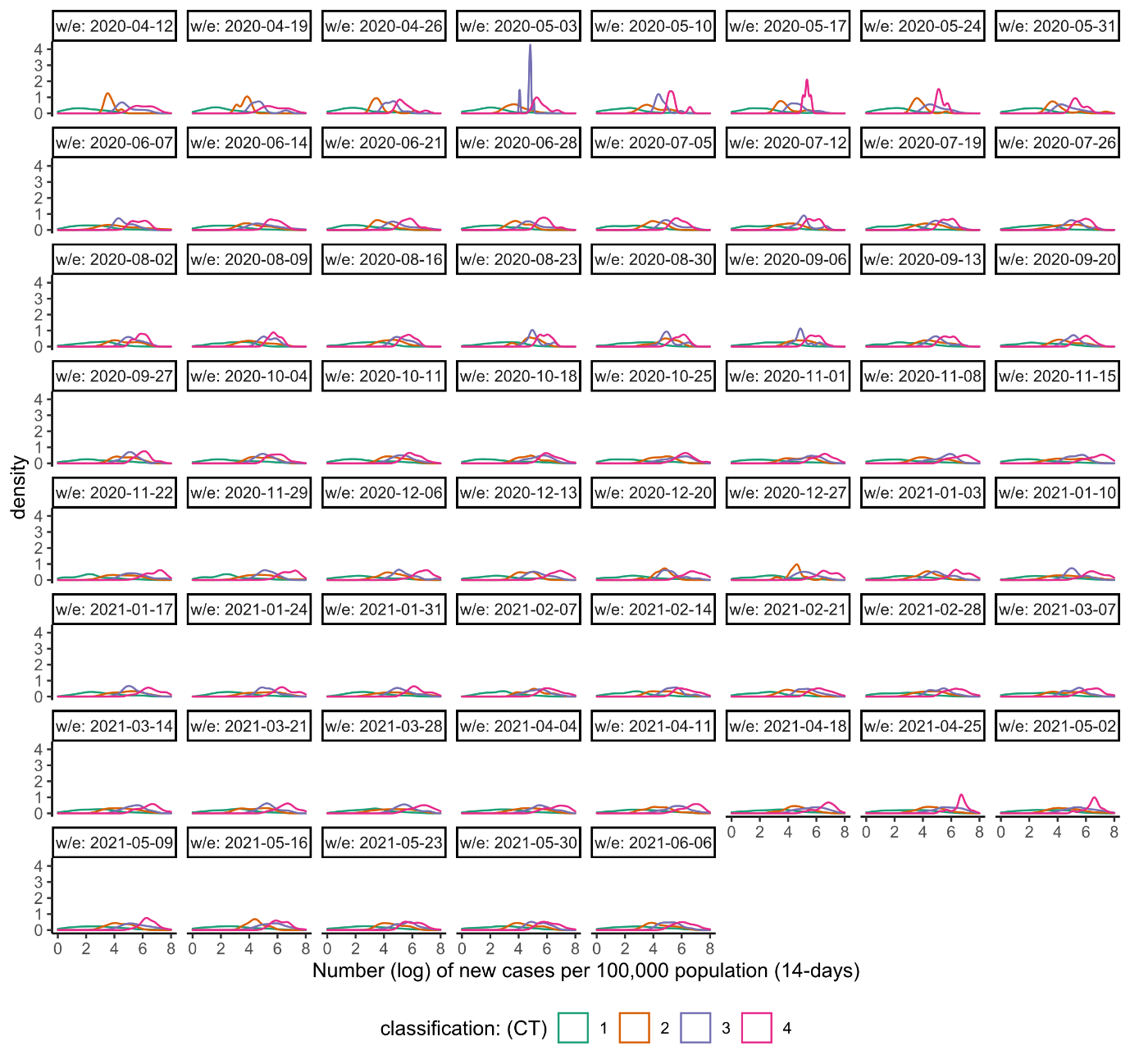


Figure 2. Density of weekly incidence rate per 100,000 population (14 days average) in each transmission class following classification guidance published in November 2020 and the median of scores as the aggregate approach.


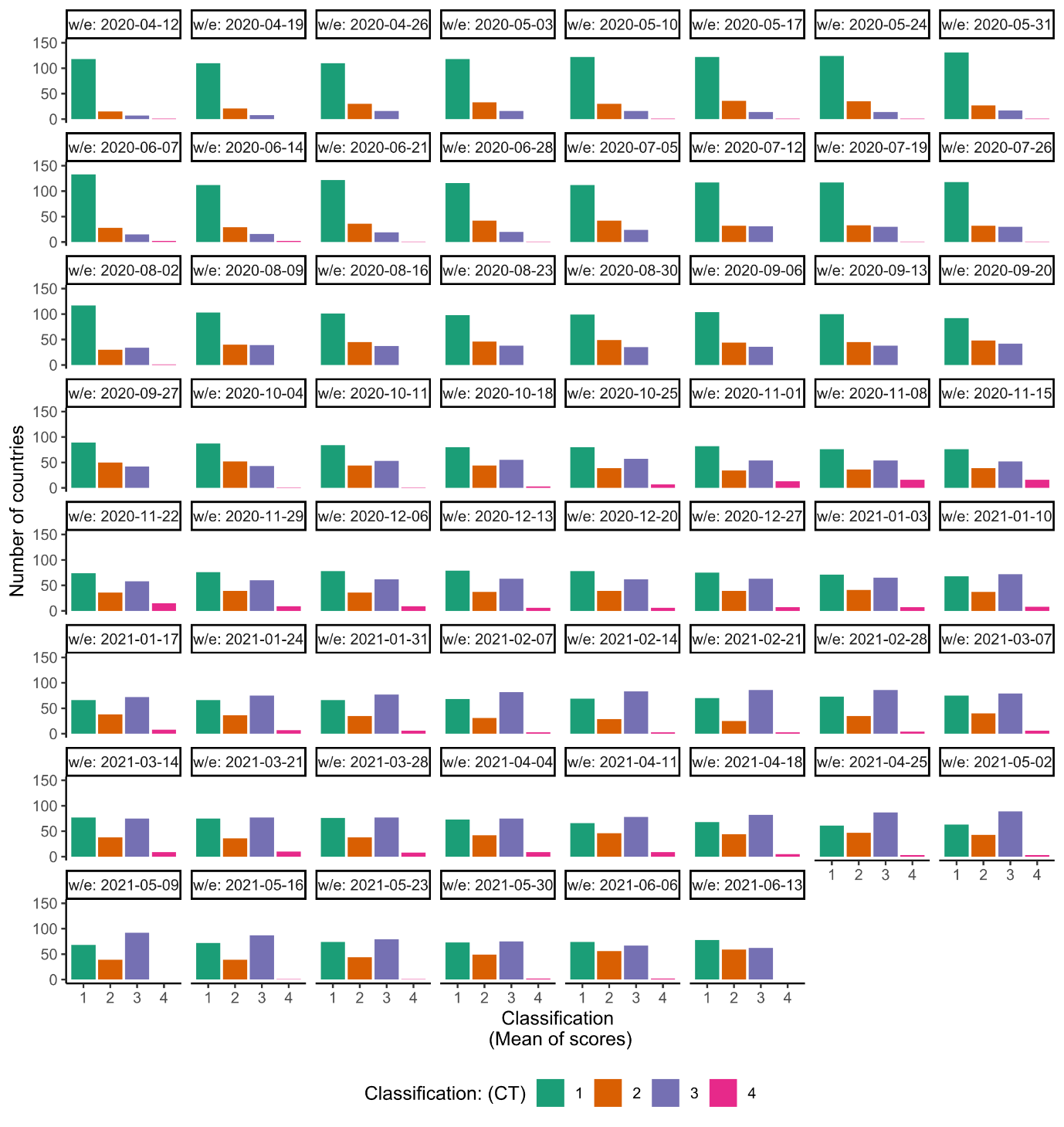


Figure 3. Frequency of country distribution according to the aggregate approach for the new classification guidelines published in November 2020 implementing the 'Mean of scores' as the aggregate criteria.


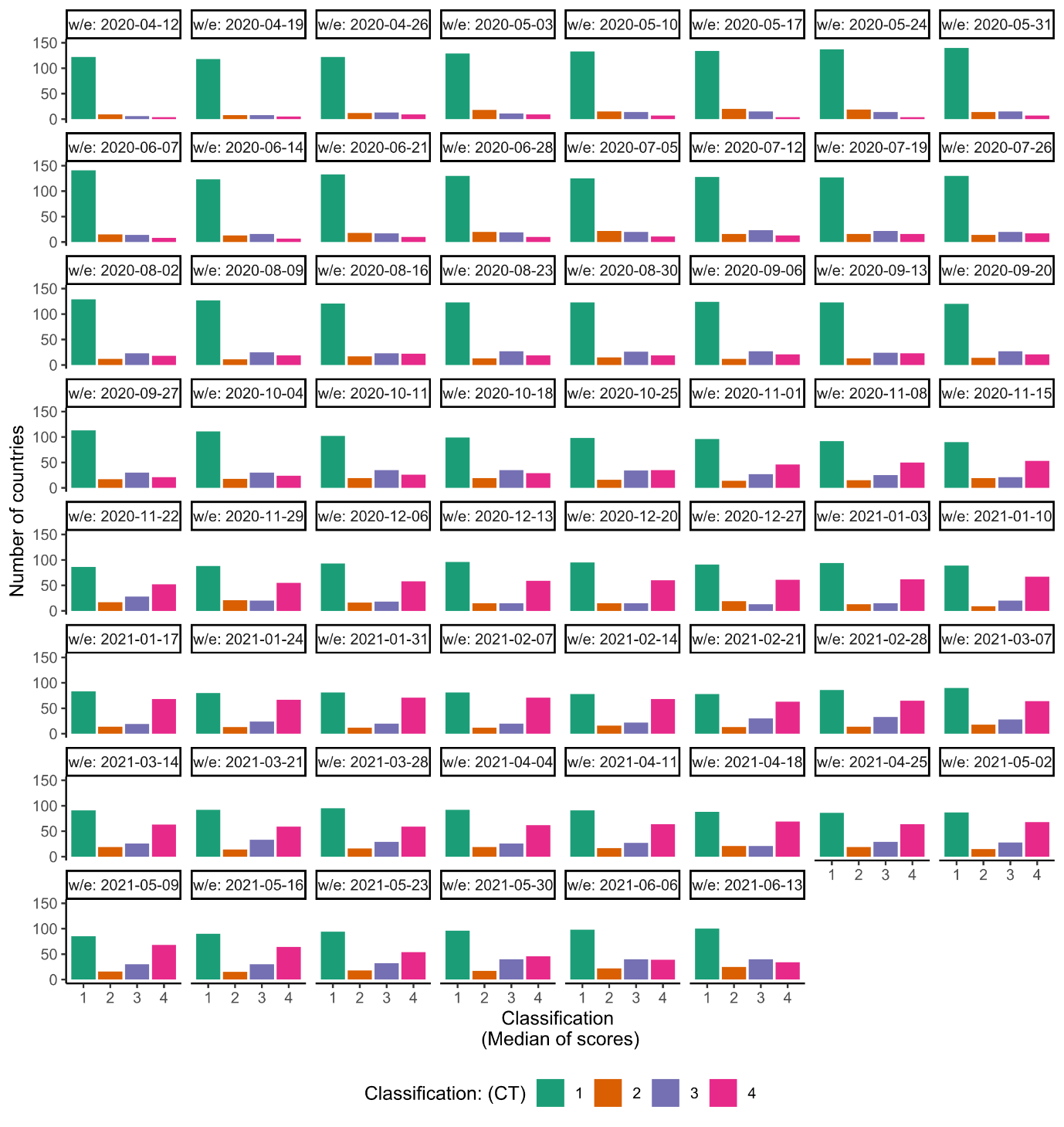


Figure 4. Frequency of country distribution according to the aggregate approach for the new classification guidelines published in November 2020 implementing the 'Median of scores' as the aggregate criteria.


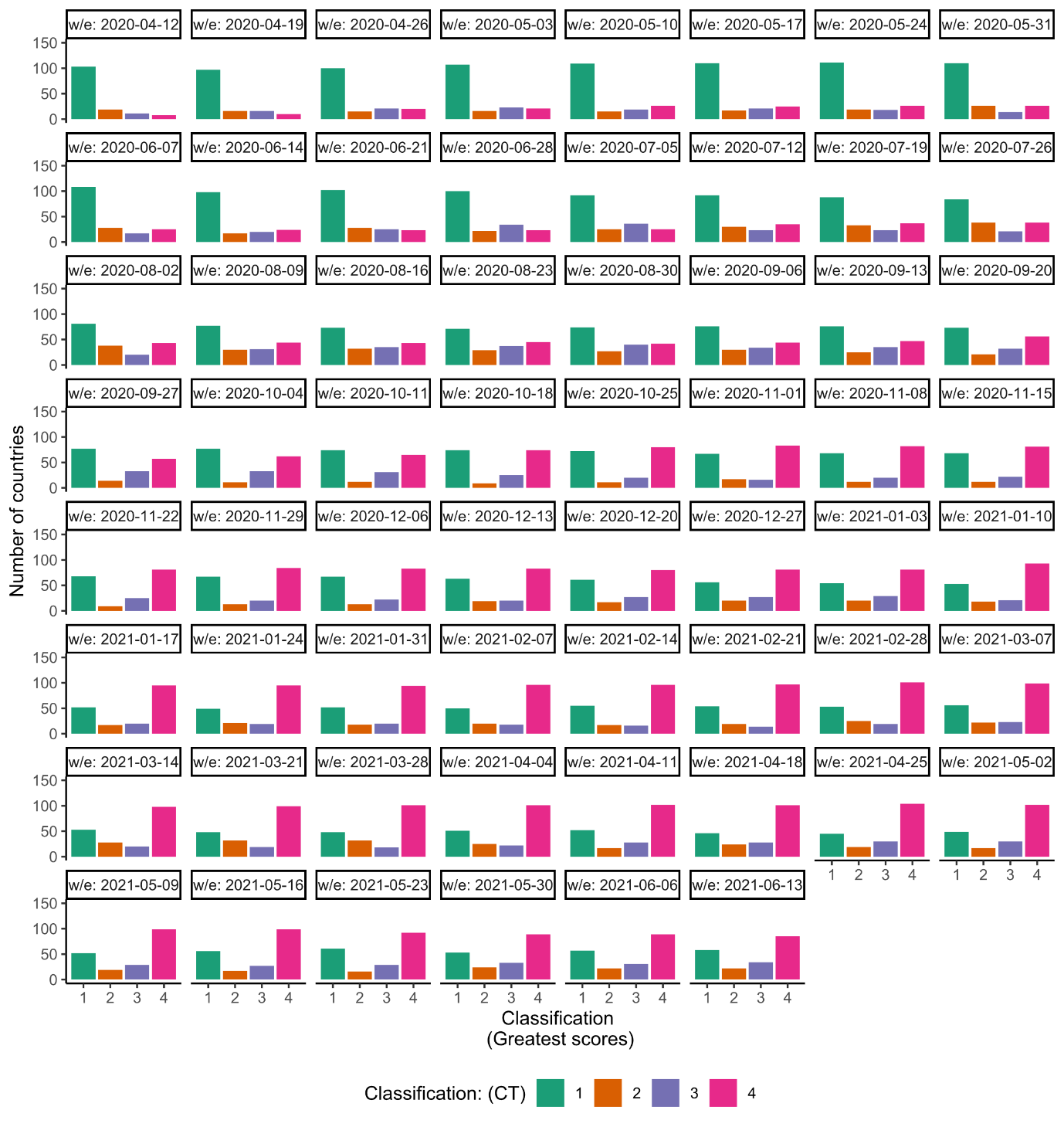


Figure 5. Frequency of country distribution according to the aggregate approach for the new classification guidelines published in November 2020 implementing the Greatest of scores' as the aggregate criteria.


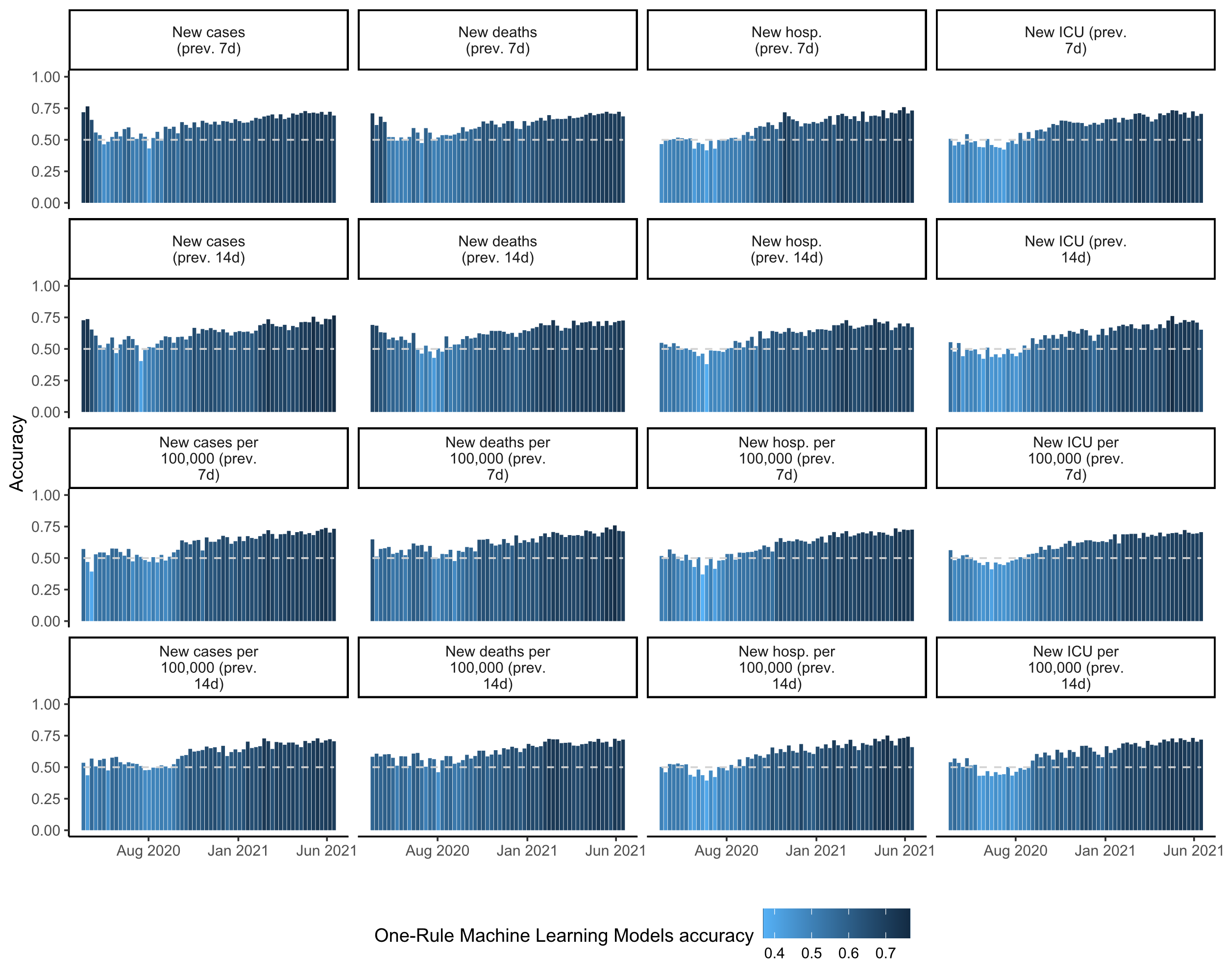

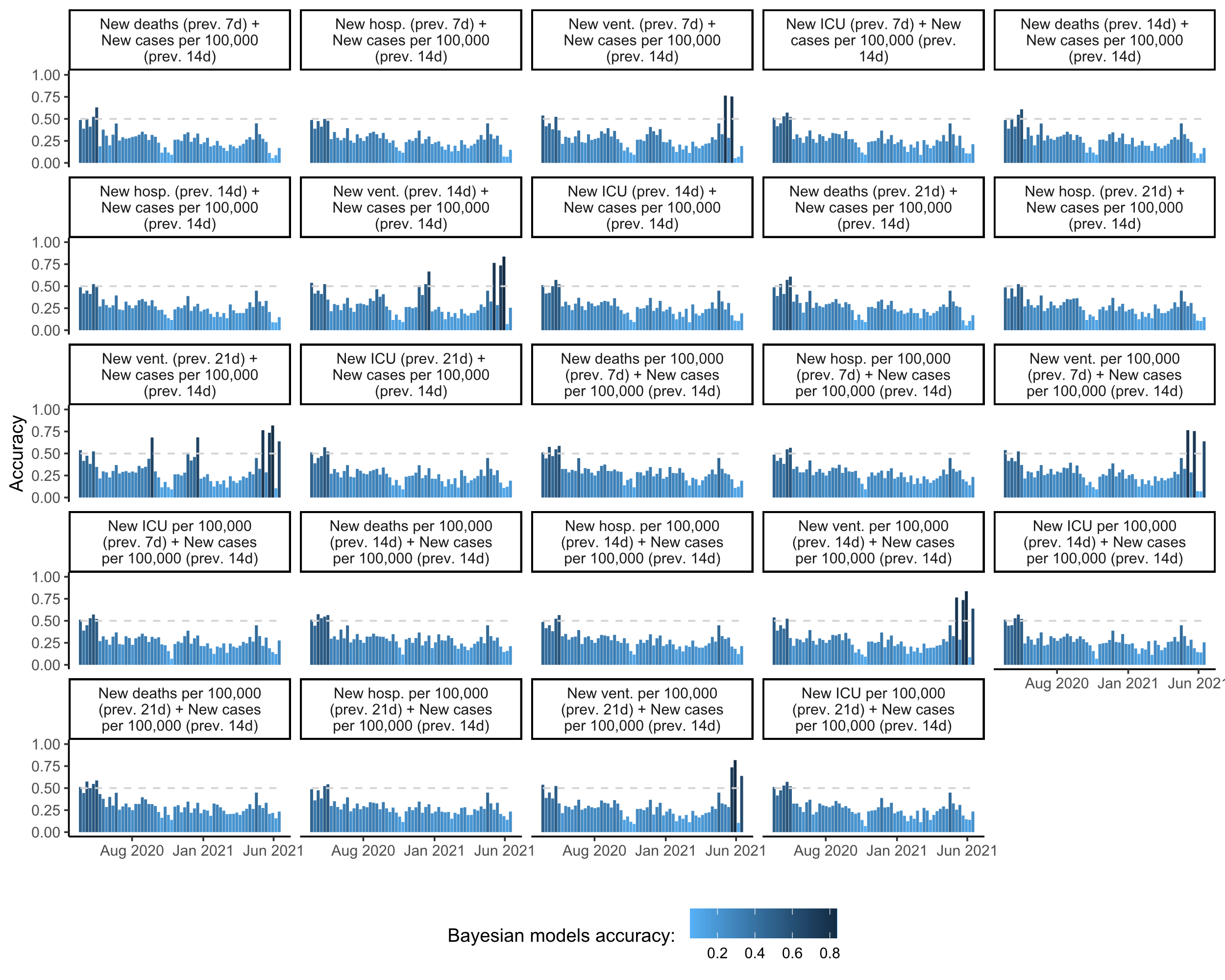


Figure 6. Predictive accuracies by week for each variable according to the initial country classification guidance published in March 2020.Each bar represents one week. Top: Bayesian models. Bottom: One-Rule Machine Learning models.


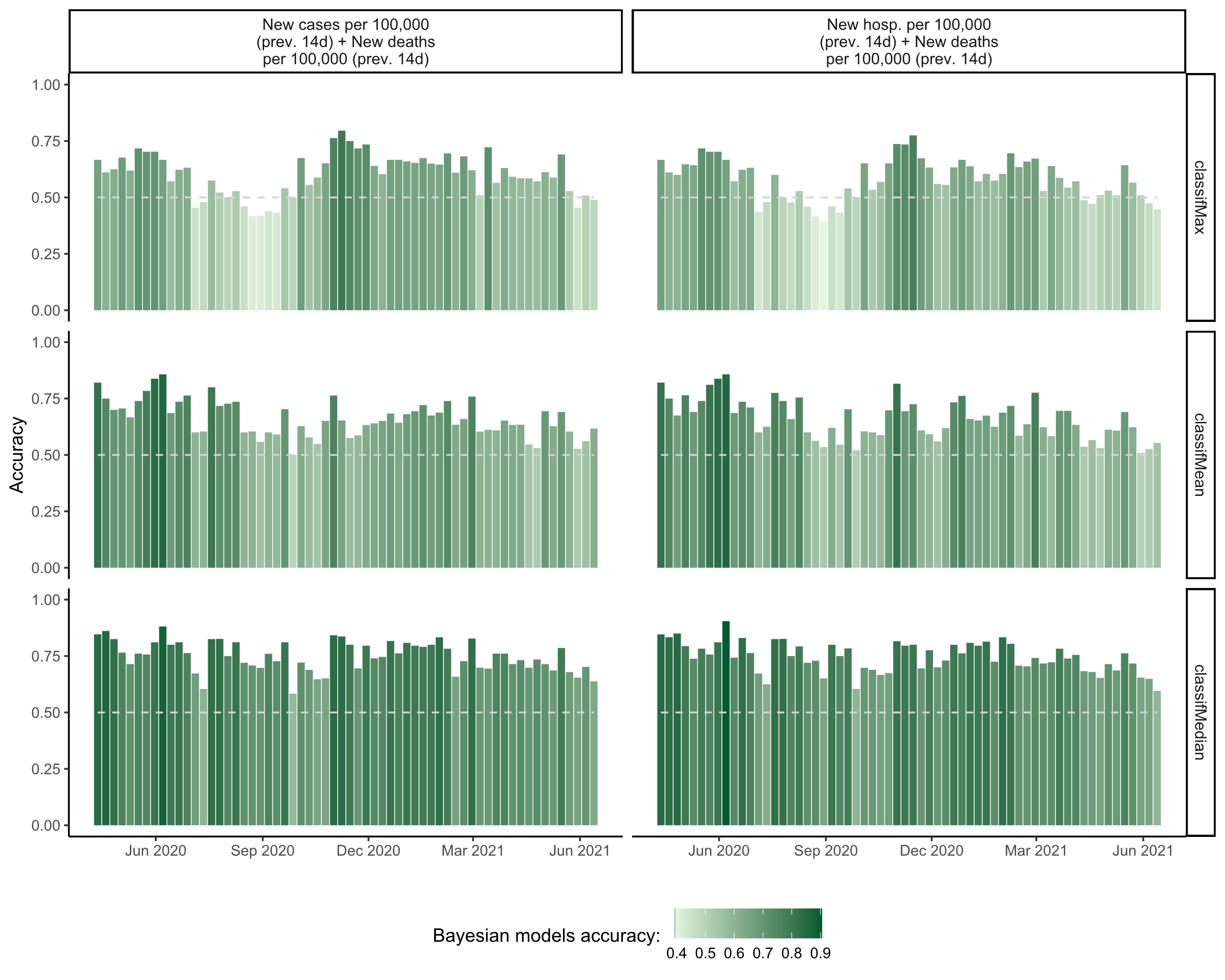

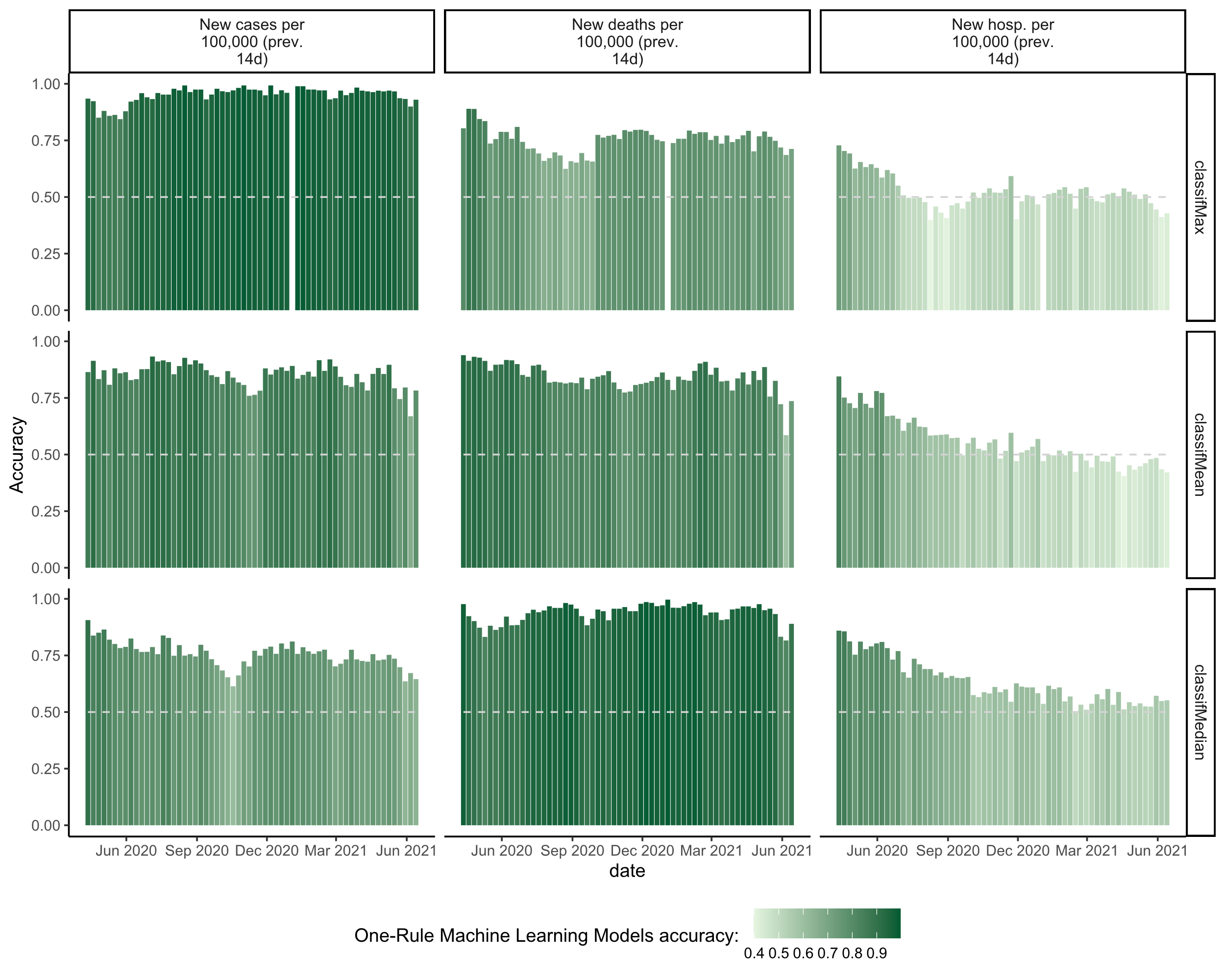


Figure 7. Predictive accuracies by week for each variable according to the update country classification guidance published in November 2020.Each bar represents one week. The variables are indicated on the upper row and labelling criteria on the right

Table 2. Summary of mixed effects ordinal regression models following the initial country classification guidance published in March 2020.

| Variable 1 | Variable 2 | Accuracy  (95% CI) |
| --- | --- | --- |
| New hosp. (14-days) | New cases per 100,000 (14-days) | 0.592  (0.574-0.610) |
| New deaths per 100,000 (14-days) | New cases per 100,000 (14-days) | 0.591  (0.573-0.610) |
| New cases | New cases per 100,000 (14-days) | 0.591  (0.573-0.609) |
| New deaths | New cases per 100,000 (14-days) | 0.591  (0.573-0.609) |
| New deaths per 100,000 (7-days) | New cases per 100,000 (14-days) | 0.591  (0.573-0.609) |
| New deaths per 100,000 (7-days) | - | 0.591  (0.574-0.609) |
| New deaths per 100,000 (14-days) | - | 0.591  (0.574-0.608) |
| New cases | - | 0.591  (0.573-0.608) |
| New deaths | - | 0. 591  (0.573-0.608) |
| New cases per 100,000 (7-days) | - | 0.591  (0.573-0.607) |

Table 3. Summary of best performing Bayesian proportional odds logistic regression models following the initial country classification guidance published in March 2020.

| Variable 1 | Variable 2 | Accuracy  (95% PI) |
| --- | --- | --- |
| New patients in ventilator (21-days) | New cases per 100,000 (14-days) | 0.323  (0.202-0.470) |
| New patients in ventilator (14-days) | New cases per 100,000 (14-days) | 0.312  (0.192-0.459) |
| New deaths per 100,000 (21-days) | New cases per 100,000 (14-days) | 0.304  (0.179-0.455) |
| New deaths per 100,000 (14-days) | New cases per 100,000 (14-days) | 0.303  (0.179-0.454) |
| New patients in ventilator per 100,000 (14-days) | New cases per 100,000 (14-days) | 0.300  (0.181-0.448) |
| New cases per 100,000 (21-days) | - | 0.259  (0.144-0.407) |
| New cases per 100,000 (14-days) | - | 0.257  (0.143-0.405) |
| New cases per 100,000 (7-days) | - | 0.256  (0.142-0.404) |
| New patients in ventilator (21-days) | - | 0.228  (0.138-0.358) |
| New patients in ventilator (14-days) | - | 0. 211  (0.123-0.341) |

Table 4. Bootstrap accuracies of best one-rule machine learning models following the initial country classification guidance published in March 2020.

| Variable | Accuracy |
| --- | --- |
| New cases (14-days) | 0.629  (0.590-0.675) |
| New cases per 100,000 (14-days) | 0.629  (0.591-0.676) |
| Cumulative deaths | 0.627  (0.585-0.670) |
| New deaths per 100,000 (14-days) | 0.626  (0.588-0.672) |

Table 5. Summary of model accuracies and 95% CI/PI for the new country classification guidance published in November 2020.

| Criteria | Variable | Mixed effects ordinal longitudinal | Proportional odds logistic regression | One-Rule Machine Learning |
| --- | --- | --- | --- | --- |
| Greatest score | New cases per 100k (14 days) | 0.535  (0·511-0·549) | 0·599  (0·445-0·739) | 0·950  (0·933-0·968) |
| Median score | New deaths per 100k (14 days) | 0·782  (0·767-0·797) | 0·741  (0·591-0·857) | 0·940  (0·919-0·960) |
| Mean score | New cases per 100k (14 days) | 0·646  (0·628-0·663) | 0·641  (0·486-0·775) | 0·852  (0·821-0·882) |
| Mean score | New deaths per 100k (14 days) | 0·515  (0·496-0·533) | 0·7023  (0·5482-0·8277) | 0·841  (0·812-0·873) |
| Median score | New cases per 100k (14 days) | 0·697  (0·680-0·714) | 0·709  (0·556-0·831) | 0·756  (0·716-0·829) |
| Greatest score | New deaths per 100k (14 days) | 0·723  (0·706-0·739) | 0·575  (0·421-0·718) | 0·752  (0·715-0·803) |

Table 6. Summary of Mixed effects ordinal longitudinal models according to the new country classification published in November 2020.

| Criteria | Variable 1 | Variable 2 | Accuracy  (95% CI) |
| --- | --- | --- | --- |
| Median score | New cases per 100k (14 days) | New deaths per 100k (14 days) | 0·823  (0·809-0·837) |
| Median score | New hosp. per 100k (14 days) | New deaths per 100k (14 days) | 0·781  (0·765-0·796) |
| Median score | New cases per 100k (14 days) | New deaths per 100k (14 days) | 0·614  (0·596-0·632) |
| Greatest score | New hosp. per 100k (14 days) | New deaths per 100k (14 days) | 0·586  (0·567- 0·604) |
| Median score | New cases per 100k (14 days) | New deaths per 100k (14 days) | 0·580  (0·561-0·598) |
| Median score | New hosp. per 100k (14 days) | New deaths per 100k (14 days) | 0·564  (0·545-0·582) |

Table 7. Agreement in predictions of pending countries between models. (Mixor: Mixed effects ordinal longitudinal model; Jags: Bayesian Proportional Odds model; OneR: 1-rule classification algorithm)

| week | w/e | Mixor & Jags | Mixor & OneR | Jags & OneR | No agreement | n |
| --- | --- | --- | --- | --- | --- | --- |
| 15 | 12 Apr 2020 | 2 | 9 | 17 | 15 | 43 |
| 16 | 19 Apr 2020 | 3 | 8 | 23 | 11 | 45 |
| 17 | 26 Apr 2020 | 2 | - | 19 | 7 | 28 |
| 18 | 03 May 2020 | - | - | 11 | 8 | 19 |
| 19 | 10 May 2020 | - | - | 10 | 7 | 17 |
| 20 | 17 May 2020 | - | - | 9 | 5 | 14 |
| 21 | 24 May 2020 | - | - | 8 | 4 | 12 |
| 22 | 31 May 2020 | - | - | 7 | 4 | 11 |
| 23 | 07 Jun 2020 | - | - | 9 | - | 9 |
| 24 | 14 Jun 2020 | - | - | 3 | 5 | 8 |
| 25 | 21 Jun 2020 | - | - | 4 | 4 | 8 |
| 26 | 28 Jun 2020 | - | - | 4 | 3 | 7 |
| 27 | 05 Jul 2020 | - | - | 4 | 2 | 6 |
| 28 | 12 Jul 2020 | - | - | 2 | 2 | 4 |
| 29 | 19 Jul 2020 | - | - | 1 | 2 | 3 |
| 30 | 26 Jul 2020 | - | - | 2 | 1 | 3 |
| 31 | 02 Aug 2020 | - | - | 1 | 1 | 2 |
| 32 | 09 Aug 2020 | - | - | 1 | 1 | 2 |
| 33 | 16 Aug 2020 | - | - | 2 | - | 2 |
| 34 | 23 Aug 2020 | - | - | 1 | 1 | 2 |
| 35 | 30 Aug 2020 | - | - | 1 | 1 | 2 |
| 36 | 06 Sep 2020 | - | - | 2 | - | 2 |
| 37 | 13 Sep 2020 | - | - | 2 | - | 2 |
| 38 | 20 Sep 2020 | - | - | 2 | - | 2 |
| 39 | 27 Sep 2020 | - | - | 2 | - | 2 |
| 40 | 04 Oct 2020 | - | - | 2 | - | 2 |
| 41 | 11 Oct 2020 | - | - | 2 | - | 2 |
| 42 | 18 Oct 2020 | - | - | 1 | 1 | 2 |
| 43 | 25 Oct 2020 | - | - | 1 | 1 | 2 |
| 44 | 01 Nov 2020 | - | - | 1 | 1 | 2 |
| 45 | 08 Nov 2020 | - | - | 1 | 1 | 2 |
| 46 | 15 Nov 2020 | - | - | 2 | - | 2 |
| 47 | 22 Nov 2020 | - | - | 2 | - | 2 |
| 48 | 29 Nov 2020 | - | - | 2 | - | 2 |
| 49 | 06 Dec 2020 | - | - | 2 | - | 2 |
| 50 | 13 Dec 2020 | - | - | 2 | - | 2 |
| 51 | 20 Dec 2020 | - | - | 1 | 1 | 2 |
| 52 | 27 Dec 2020 | - | - | 1 | 1 | 2 |
| 53 | 03 Jan 2021 | - | - | 1 | 1 | 2 |
| 1 | 10 Jan 2021 | - | - | 2 | - | 2 |
| 2 | 17 Jan 2021 | - | - | 3 | - | 3 |
| 3 | 24 Jan 2021 | - | - | 3 | - | 3 |
| 4 | 31 Jan 2021 | - | - | 3 | - | 3 |
| 5 | 07 Feb 2021 | - | - | 3 | - | 3 |
| 6 | 14 Feb 2021 | - | - | 3 | - | 3 |
| 7 | 21 Feb 2021 | - | - | 3 | - | 3 |
| Total |  | 7 | 17 | 188 | 91 | 303 |

**Confusion Matrices Initial classification (March 2020):**

**Model: Ordinal Longitudinal Regression model.**

**Predictor: New deaths per 100k (7-days).**

Table 8. Confusion matrix of model Ordinal Longitudinal Regression with one predictor (New deaths per 100k (7-days)) following Initial Classification (March 2020).

| Actual classification: | "Sporadic Cases" | "Clusters of Cases" | "Community Transmission" |
| --- | --- | --- | --- |
| Predicted classification: |  |  |  |
| " Sporadic Cases " | 0 | 0 | 1 |
| " Clusters of Cases " | 0 | 0 | 0 |
| " Community Transmission " | 314 | 849 | 1683 |

**Ordinal Longitudinal Regression model.**

**Predictors: New hosp. (14-days) and New cases per 100k (14-days).**

Table 9. Confusion matrix of model Ordinal Longitudinal Regression with two predictors (New hosp. (14-days) and New cases per 100k (14-days)) following Initial Classification (March 2020).

| Actual classification: | "Sporadic Cases" | "Clusters of Cases" | "Community Transmission" |
| --- | --- | --- | --- |
| Predicted classification: |  |  |  |
| "Sporadic Cases" | 0 | 0 | 1 |
| "Clusters of Cases" | 0 | 0 | 8 |
| "Community Transmission" | 314 | 849 | 1675 |

**Proportional Odds Model (Bayesian).**

**Predictor: New cases per 100k (14-days). Initial classification (March 2020).**

Table 10. Confusion matrix of Proportional Odds Model (Bayesian) with one predictor (New cases per 100k (14-days)) following Initial Classification (March 2020).

| Actual classification: | "Sporadic Cases" | "Clusters of Cases" | "Community Transmission" |
| --- | --- | --- | --- |
| Predicted classification: |  |  |  |
| "Sporadic Cases" | 149 | 49 | 116 |
| "Clusters of Cases" | 427 | 94 | 328 |
| "Community Transmission" | 1143 | 60 | 481 |

**Proportional Odds Model (Bayesian).**

**Predictors:** **New cases per 100k (14-days) and New patients on ventilator (21-days). Initial classification (March 2020).**

Table 11. Confusion matrix of Proportional Odds Model (Bayesian) with two predictors (New cases per 100k (14-days) and New patients on ventilator (21-days)) following Initial Classification (March 2020).

| Actual classification: | "Sporadic Cases" | "Clusters of Cases" | "Community Transmission" |
| --- | --- | --- | --- |
| Predicted classification: |  |  |  |
| "Sporadic Cases" | 127 | 49 | 138 |
| "Clusters of Cases" | 377 | 106 | 366 |
| "Community Transmission" | 923 | 58 | 703 |

**One-Rule classification model.**

**Predictor: New deaths (14-days). Initial classification (March 2020).**

Table 12. Confusion matrix of One-Rule classification model with one predictor (New deaths (14-days)) following Initial Classification (March 2020).

| Actual classification: | "Sporadic Cases" | "Clusters of Cases" | "Community Transmission" |
| --- | --- | --- | --- |
| Predicted classification: |  |  |  |
| "Sporadic Cases" | 113 | 35 | 166 |
| "Clusters of Cases" | 74 | 159 | 616 |
| "Community Transmission" | 46 | 116 | 1522 |

**Confusion Matrices. New classification (November 2020):**

**Ordinal Longitudinal Regression model. Predictor: New deaths per 100k (14-days). Aggregate criteria: Median score.**

Table 13. Confusion matrix of model Ordinal Longitudinal Regression with one predictor (New deaths per 100k (14-days) and median aggregate criteria) following New Classification (November 2020).

| Actual classification: | "CT-1" | " CT-2" | " CT-3" | " CT-4" |
| --- | --- | --- | --- | --- |
| Predicted classification: |  |  |  |  |
| "CT-1" | 1588 | 256 | 98 | 2 |
| "CT-2" | 6 | 3 | 97 | 0 |
| "CT-3" | 3 | 9 | 159 | 114 |
| "CT-4" | 0 | 2 | 33 | 477 |

**Ordinal Longitudinal Regression model. Predictors: New hosp. per 100k (14 days) and New cases per 100k (14 days). Aggregate criteria: Median score.**

Table 14. Confusion matrix of model Ordinal Longitudinal Regression with two predictors (New hosp. per 100k (14-days) and New cases per 100k (14-days)) following New Classification (November 2020).

| Actual classification: | "CT-1" | " CT-2" | " CT-3" | " CT-4" |
| --- | --- | --- | --- | --- |
| Predicted classification: |  |  |  |  |
| "CT-1" | 1577 | 217 | 24 | 0 |
| "CT-2" | 13 | 35 | 105 | 0 |
| "CT-3" | 7 | 14 | 221 | 82 |
| "CT-4" | 0 | 4 | 37 | 511 |

**Proportional Odds Model (Bayesian). Predictor:** **New deaths per 100k (14 days). Aggregate criteria: Median score.**

Table 15. Confusion matrix of Proportional Odds Model (Bayesian) with one predictors (New deaths per 100k (14 days)) following New Classification (November 2020).

| Actual classification: | "CT-1" | " CT-2" | " CT-3" | " CT-4" |
| --- | --- | --- | --- | --- |
| Predicted classification: |  |  |  |  |
| "CT-1" | 1248 | 198 | 225 | 15 |
| "CT-2" | 0 | 0 | 3 | 0 |
| "CT-3" | 0 | 2 | 13 | 52 |
| "CT-4" | 0 | 0 | 11 | 295 |

**Proportional Odds Model (Bayesian). Predictors: New cases per 100k (14 days) and New deaths per 100k (14 days). Aggregate criteria: Median score.**

Table 16. Confusion matrix of Proportional Odds Model (Bayesian) with two predictors (New cases per 100k (14 days) and New deaths per 100k (14 days)) following New Classification (November 2020)

| Actual classification: | "CT-1" | " CT-2" | " CT-3" | " CT-4" |
| --- | --- | --- | --- | --- |
| Predicted classification: |  |  |  |  |
| "CT-1" | 1592 | 0 | 2 | 3 |
| "CT-2" | 265 | 0 | 3 | 2 |
| "CT-3" | 303 | 1 | 44 | 39 |
| "CT-4" | 19 | 0 | 81 | 493 |

**One-Rule classification model. Predictor: New cases per 100k (14 days). Aggregate criteria: Greatest score. New classification (November 2020).**

Table 17. Confusion matrix of One-Rule classification model with one predictor (New cases per 100k (14-days)) following New Classification (November 2020).

| Actual classification: | "CT-1" | " CT-2" | " CT-3" | " CT-4" |
| --- | --- | --- | --- | --- |
| Predicted classification: |  |  |  |  |
| "CT-1" | 1043 | 20 | 0 | 0 |
| "CT-2" | 16 | 281 | 23 | 0 |
| "CT-3" | 12 | 19 | 339 | 30 |
| "CT-4" | 2 | 2 | 39 | 1021 |

**Comparison of model predictions between initial and updated classifications:**

Table 18. McNemar tests comparison of predictions between initial classification (March 2020) models and new classification (November 2020) models.

| **Ordinal Longitudinal Regression model**  **(univariate)** | Initial classification | |  |  |
| --- | --- | --- | --- | --- |
| New classification | Correct | Incorrect | McNemar's chi-squared | p-value |
| Correct | 1336 | 891 | 237.1 | < 2.2e-16 |
| Incorrect | 348 | 272 |  |  |
|  |  |  |  |  |
| **Ordinal Longitudinal Regression model**  (two predictors) | Initial classification | |  |  |
| New classification | Correct | Incorrect |  |  |
| Correct | 1363 | 981 | 345.11 | < 2.2e-16 |
| Incorrect | 312 | 191 |  |  |
|  |  |  |  |  |
| **Proportional Odds Model (Bayesian).**  (univariate) | Initial classification | |  |  |
| New classification | Correct | Incorrect |  |  |
| Correct | 641 | 1459 | 1226.1 | < 2.2e-16 |
| Incorrect | 83 | 664 |  |  |
|  |  |  |  |  |
| **Proportional Odds Model (Bayesian).**  (two predictors) | Initial classification | |  |  |
| New classification | Correct | Incorrect |  |  |
| Correct | 775 | 1354 | 937.86 | < 2.2e-16 |
| Incorrect | 161 | 557 |  |  |
|  |  |  |  |  |
| **One-Rule classification model** | Initial classification | |  |  |
| New classification | Correct | Incorrect |  |  |
| Correct | 1693 | 991 | 723.74 | < 2.2e-16 |
| Incorrect | 101 | 62 |  |  |

**Pairwise comparisons of updated classification (November 2020) models:**

Table 19. Pairwise McNemar tests comparison of predictions between models following updated classification (November 2020).

|  | **One-Rule classification model** | |  |  |
| --- | --- | --- | --- | --- |
| **Proportional Odds Model (Bayesian).** | Correct | Incorrect | McNemar's chi-squared | p-value |
| Correct | 2044 | 85 | 423.33 | < 2.2e-16 |
| Incorrect | 640 | 78 |  |  |
|  |  |  |  |  |
|  | **Ordinal Longitudinal Regression model** | |  |  |
| **Proportional Odds Model (Bayesian).** | Correct | Incorrect | McNemar's chi-squared | p-value |
| Correct | 1763 | 366 | 48.36 | 3.549e-12 |
| Incorrect | 581 | 137 |  |  |
|  |  |  |  |  |
|  | **One-Rule classification model** | |  |  |
| **Ordinal Longitudinal Regression model** | Correct | Incorrect | McNemar's chi-squared | p-value |
| Correct | 2217 | 127 | 193.47 | < 2.2e-16 |
| Incorrect | 467 | 36 |  |  |
